# Point-of-Care Surface Plasmon Resonance Biosensor for Stroke Biomarkers NT-proBNP and S100β Using a Functionalized Gold Chip with Specific Antibody

**DOI:** 10.3390/s19112533

**Published:** 2019-06-03

**Authors:** Dorin Harpaz, Brescia Koh, Robert S. Marks, Raymond C.S. Seet, Ibrahim Abdulhalim, Alfred I.Y. Tok

**Affiliations:** 1 School of Material Science & Engineering, Nanyang Technology University, 50 Nanyang Avenue, Singapore 639798, Singapore; DORIN001@e.ntu.edu.sg (D.H.); rkoh009@e.ntu.edu.sg (B.K.); 2 Department of Biotechnology Engineering, Ben-Gurion University of the Negev, Beer-Sheva 84105, Israel; rsmarks@bgu.ac.il; 3 Institute for Sports Research (ISR), Nanyang Technology University and Loughborough University, Nanyang Avenue, Singapore 639798, Singapore; 4 The National Institute for Biotechnology in the Negev, Ben-Gurion University of the Negev, Beer-Sheva 84105, Israel; 5 The Ilse Katz Centre for Meso and Nanoscale Science and Technology, Ben-Gurion University of the Negev, Beer-Sheva 84105, Israel; 6 Division of Neurology, Department of Medicine, Yong Loo Lin School of Medicine, National University of Singapore, 10 Medical Dr, Singapore 117597, Singapore; mdcrscs@nus.edu.sg; 7 Department of Electro optics and Photonics Engineering, Ben Gurion University of the Negev, Beer-Sheva 84105, Israel

**Keywords:** surface-plasmon-resonance, refractive-index, point-of-care, biosensor, stroke, diagnostics, biomarkers, NT-proBNP, S100β, Au SPR-chip

## Abstract

Surface-plasmon-resonance (SPR) is a quantum-electromagnetic phenomenon arising from the interaction of light with free electrons at a metal-dielectric interface. At a specific angle/wavelength of light, the photon’s energy is transferred to excite the oscillation of the free electrons on the surface. A change in the refractive-index (RI) may occur, which is influenced by the analyte concentration in the medium in close contact with the metal surface. SPR has been widely used for the detection of gaseous, liquid, or solid samples. In this study, a functionalized specific SPR chip was designed and used in a novel point-of-care SPR module (PhotonicSys SPR H5) for the detection of the stroke biomarkers NT-proBNP and S100β. These biomarkers have proven to be good for stroke diagnosis, with sensitivity and specificity of >85%. Specific detection was done by binding a biomolecular-recognizing antibody onto the Au SPR-chip. Detection was tested in water and plasma samples. NT-proBNP and S100β were detected in a range of concentrations for stroke, from 0.1 ng/mL to 10 ng/mL. The RI of the blank plasma samples was 1.362412, and the lowest concentration tested for both biomarkers showed a prominent shift in the RI signal (0.25 ng/mL NT-proBNP (1.364215) and S100β (1.364024)). The sensor demonstrated a clinically relevant limit-of-detection of less than ng/mL.

## 1. Introduction

**Figure 1:**
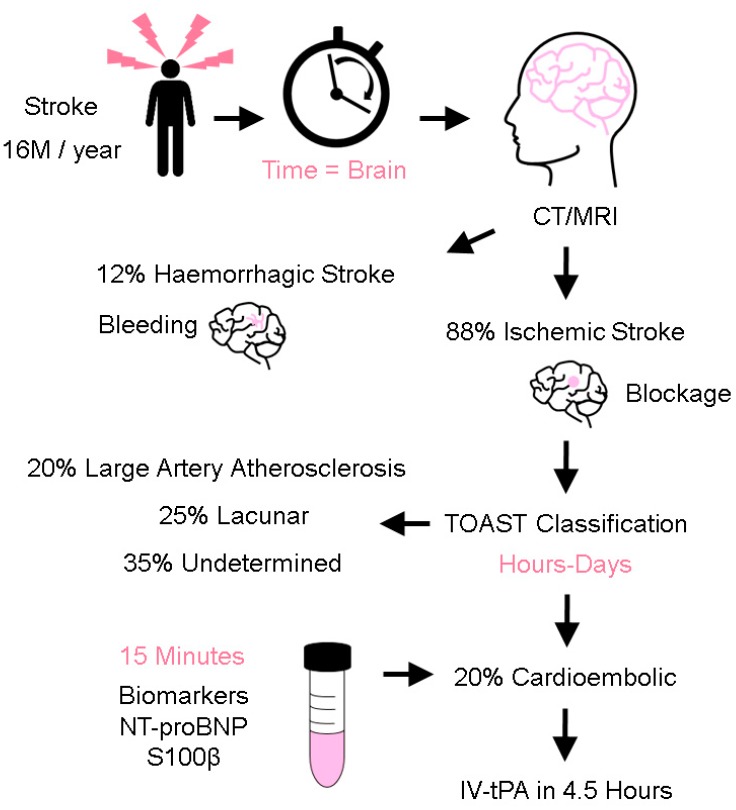
Stroke Triaging. After stroke occurrence, Time = Brain. Efficacy of care is correlated to a timely diagnosis and treatment. First, a CT/MRI scan is conducted to differentiate between an ischemic and hemorrhagic stroke. This is followed by stroke mechanisms (e.g., using the criteria of Trial of Org 10172 in Acute Stroke Treatment, TOAST classification) to identify a cardioembolic stroke. Patients with cardioembolic stroke harbor a higher risk of poorer stroke outcomes and timely treatment with acute reperfusion treatment is necessary (intravenous tissue plasminogen activator (IV-tPA)) within the time-limited window of 4.5 h. The ability to accurately measure stroke biomarkers (e.g., NT-proBNP and S100β) within 15 min is highly desirable, in order to shorten the classification time.

Stroke is the second leading cause of death globally [1] and is a costly disease from a human, economic, and societal perspective [2]. Annually, there are more than 16 million stroke cases, and an estimated 6.7 million people die from stroke globally [3]. Given that age is a significant risk factor for stroke, the burden of stroke is likely to increase further as the global population ages. The efficacy of stroke treatment is highly dependent on a timely diagnosis and administration of treatment [4,5]. It is thought that approximately 1.9 million neurons die every minute following the onset of cerebral ischemia [6]. Triage of patients into two stroke mechanisms (ischemic and hemorrhagic) is important as their treatment is markedly different; treatment of patients with ischemic stroke centers around fibrinolytic drugs that, when administered in wrongly-selected patients, could aggravate the extent of bleeding in patients with a hemorrhagic stroke (Figure 1). Ischemic stroke accounts for approximately 88% of stroke cases and is further classified into cardioembolism, large artery atherosclerosis, lacunar, and undetermined causes [7,8]. Cardioembolic strokes are generally more severe, prone to reoccurrence, and account for 20% of ischemic stroke cases. Early recognition of stroke mechanisms is important to explain how strokes occur and to select appropriate secondary stroke prevention treatment. As patients with cardioembolic stroke tend to do poorly, prompt treatment with acute reperfusion strategies is necessary to arrest the initial cascade and downstream consequences of cerebral ischemia. Current methods of reperfusion treatment center around the use of intravenous recombinant tissue plasminogen activator (IV-tPA) and endovascular thrombectomy to either lyse, retrieve, or aspirate the culprit thrombus [9]. Existing methods to elucidate stroke mechanisms rely on the use of clinical examination and information derived from neuroimaging investigations such as CT and MRI brain scans [10]. Despite extensive investigations, stroke mechanisms could still not be determined in ~35% of patients, highlighting the biological complexity of stroke pathophysiology. Existing methods to elucidate stroke mechanisms can take days to weeks, depending on the timely access and availability of neuroimaging, prolonged electrocardiographic, and transesophageal echocardiogram investigations [11,12]. 

Studies have suggested that the diagnosis of stroke can be complemented with the use of blood-based biomarker measurements, thus saving significant time and adding accuracy [13]. Biomarker, a portmanteau of “biological marker”, is the objective indicator of a biological state [14]. Biomarkers should ideally be reproducible and signal the occurrence of a medical condition even before symptoms appear. Several biomarkers were tested for their usefulness as a blood-based diagnostic tool for stroke [15]. Two candidate biomarkers that have been extensively studied and have shown great diagnostic accuracy are the brain natriuretic peptide (NT-proBNP) and S100β. Recent studies have shown that elevated plasma NT-proBNP [9,16,17,18,19,20,21,22,23,24,25,26,27,28,29] and S100β [30,31,32,33,34,35,36] levels are predictive of cardioembolic stroke. NT-proBNP, a biologically inactive 76-amino acid peptide [37], is the derivative of brain natriuretic peptide (BNP) [38], a neurohormone produced mainly by the heart ventricles [39] in response to excessive stretching of cardiomyocytes (heart muscle cells) [40]. NT-proBNP has a longer half-life and higher molecular weight (Mw: 8457.4 Da; half-life: 120 min) than BNP (Mw: 3466 Da; half-life: 20 min) [41]. Therefore, most trials prefer to use NT-proBNP as a biomarker for stroke detection. S100β is among the family of non-ubiquitous Ca^2+^-modulated proteins and it is mainly produced by astrocytes. It plays an important role in nerve growth, differentiation, and reparation of nerves [42,43,44]. It is a glial protein, with a molecular weight of approximately 10 kDa, located intracellularly [45] and found in abundance in the cerebral astroglial compartment, peripheral Schwann cells, and extraneuronally in melanocytes, adipocytes, and chondrocytes [46]. Our ongoing study (not yet published) on 600 stroke patients in Singapore showed that NT-proBNP and S100β were the best predictors for cardioembolic stroke (out of 30 different biomarkers). Data mining analysis showed that plasma levels of NT-proBNP ≥ 0.5 ng/mL and S100β ≥ 0.1 ng/mL are associated with cardioembolic stroke, with a sensitivity and a specificity higher than 85%. The cut-off values for both biomarkers set a need for a highly sensitive biosensor that can measure the biomarkers in concentrations less then ng/mL.

There is a need for a rapid and portable point-of-care (POC) device, which can differentiate cardioembolic stroke from others, as a complementary tool in common clinical practice. The biosensor platform will enable faster diagnosis and a quicker response time that may improve patient survival, reduced hospitalization time, and provide significant healthcare savings. Thus far, several POC devices were tested for their usefulness in stroke prognosis [1]. These POC biosensors use electrochemical, optical, fluorescent, and luminescent-based technologies. However, there has been no attempt to develop a surface plasmon resonance (SPR)-based POC biosensor for a rapid and objective bedside test for the stroke patient [47]. SPR is a quantum electromagnetic phenomenon arising from the interaction of light with free electrons at a metal-dielectric interface [48]. At a specific angle or wavelength of light, the photon’s energy is transferred to excite the oscillation of the free electrons on the surface [49]. The excited free electrons oscillation formed at the surface is called surface plasmons (SPs). The SPs are strongly localized across the interface and may be considered as electromagnetic surface waves that propagate along the interface between a dielectric and a metal, and evanescent along the direction perpendicular to the interface [48,49]. Localized SPs (LSPs), which are usually excited from free space on the surface of metal nanoparticles, are differentiated from extended SPs (ESPs), which have a longer propagation length and stronger sensitivity to the refractive index of the environment. The interaction between LSPs and ESPs is of interest for biosensing and other applications due to the ultra-high local field enhancement [50]. The extended SPR phenomenon causes a dip in reflectance at that specific wavelength or angle due to the absorption of the photon energy in the metal. In the case of exciting ESPs using nano-perforated metals such as periodic nanoslit, a transmission peak is observed in what is called extraordinary or enhanced optical transmission [51]. The wave vector of SPs at a metal-dielectric interface is larger than that of the light wave in the dielectric [49] and this prevents SPs from exciting when a direct light wave is incident on the interface. To allow optical excitation of SPs, the wave vector of the incident light must be increased by passing the light wave through a diffracting structure or an optically denser medium via attenuated total reflection in a prism configuration at an angle larger than the critical angle. The configuration of the coupler needed to fulfill this includes a prism, grating, fiber, or waveguide coupling. In an SPR sensor, prism coupling is favorable for the development of simple miniaturized multichannel sensing devices on a single chip [52]. The wave on the interface is exceptionally sensitive to changes in the RI of the medium adjacent to the metal surface within the range of the SP field [53]. These changes may result in a shifting of the incident light’s resonant wavelength, a change in reflected light intensity [54], a change in incident light’s resonant angle [55], or a change in the phase of the reflected wave [56]. SPR has been widely used for the detection of gaseous, liquid, or solid samples [57]. 

**Figure 2:**
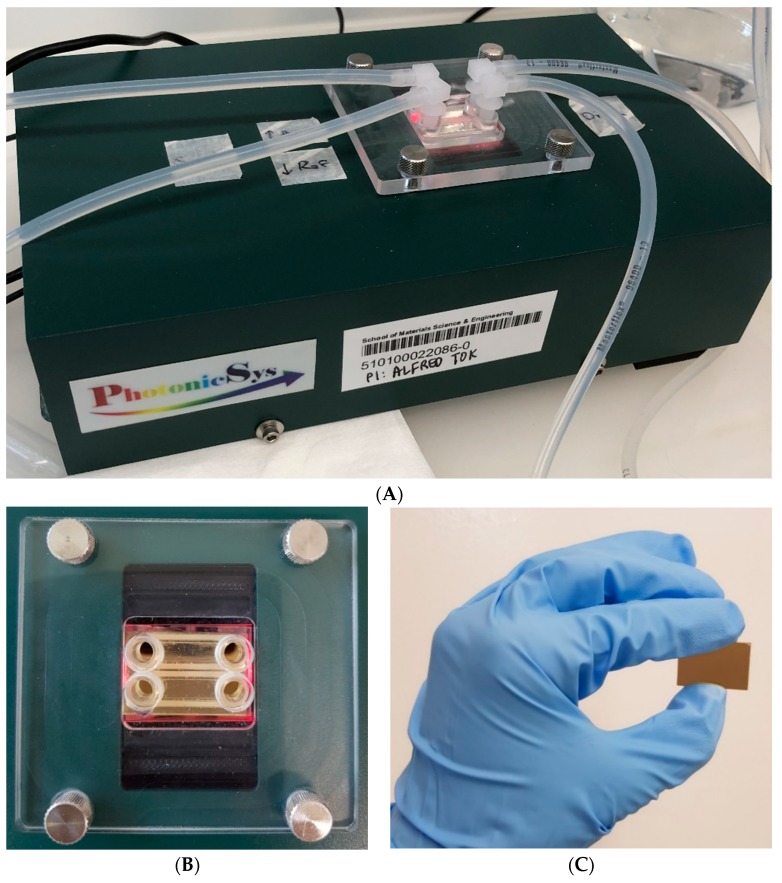
Point-of-care surface plasmon resonance biosensor. (**A**) The surface plasmon resonance biosensor is 5 cm tall and weighs 1 kg, the measurement is conducted with a connection to a pump. (**B**) A two-channel structure on top of the surface-plasmon-resonance (SPR) gold chip. (**C**) The SPR gold chip (25 mm × 15 mm × 1 mm).

**Figure 3:**
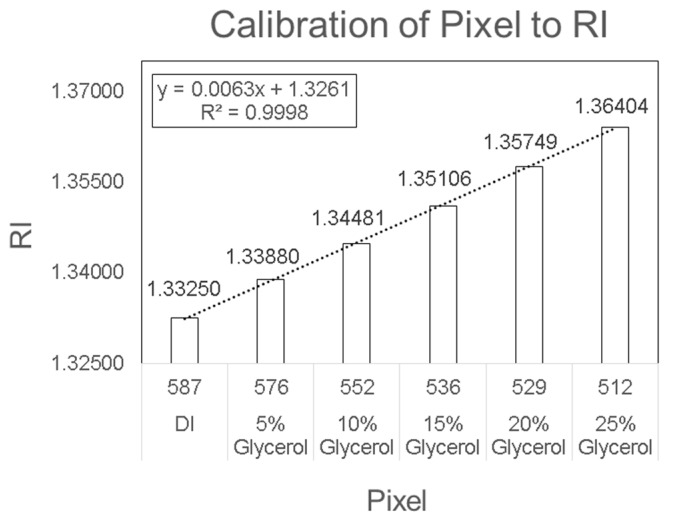
Biosensor calibration of pixel to refractive index.

**Figure 4:**
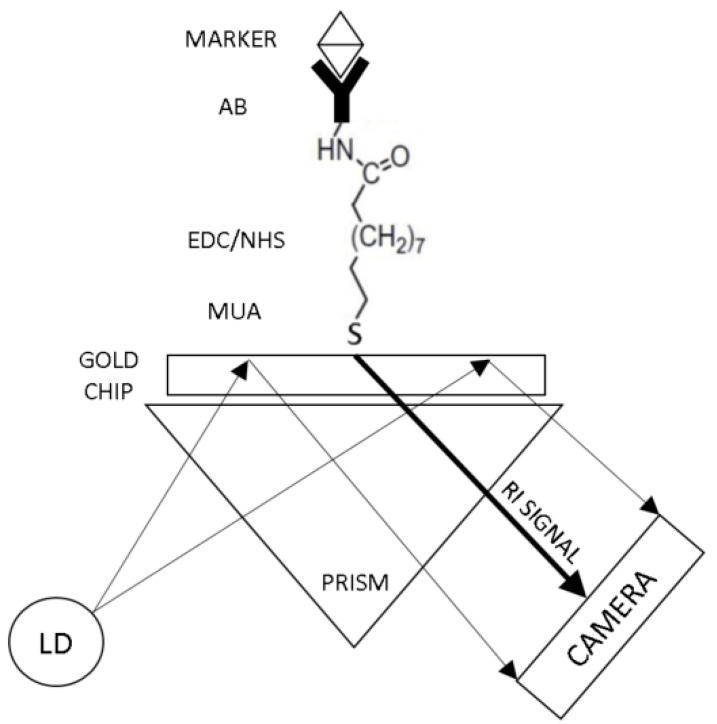
Gold chip for surface plasmon resonance biosensor. The gold chip was functionalized with a specific antibody based on MUA-EDC/NHS reaction (MUA: 11-Mercaptoundecanoic acid; EDC: N-(3-Di-methylaminopropyl)-N′-ethyl carbodiimide hydrochloride; NHS: N-Hydroxysuccinimide).

In this study, a functionalized SPR chip is developed for the specific detection of stroke biomarkers using an SPR POC device. This is the first time SPR technology is utilized as a POC device for the detection of blood-based biomarkers. SPR sensing technology is well known for its high detection sensitivity, which is required for the measurement of stroke biomarkers. However, due to its bulky setup and complicated signal acquisition, SPR has never been utilized as a POC device. This study shows the use of a novel SPR module (PhotonicSys SPR H5, Figure 2) that is tested for the detection of the stroke biomarkers NT-proBNP and S100β. This miniature SPR module is portable and can be easily integrated with other instruments, making it attractive as a POC sensor. The novelty of this SPR technology arises from its ability to measure sub-ng/mL concentrations of blood-based stroke biomarkers for point-of-care tests. The SPR biosensor mechanism is based on the detection of biomarkers using a functionalized SPR chip. The SPR chip is functionalized with a bio-specific capture entity (antibody), which acts as the biomolecular recognition element [49]. When a concentration of the biomarker is inserted onto the biosensor, the biomolecular interaction of specific binding of the analyte is detected as a RI change on the sensor surface. The overall performance of the SPR-based biosensor depends on both the characteristics of the surface functionalization as well as the optical performance of the SPR sensor. The fact that the optical field is evanescent, i.e., penetrates only to a small distance (usually few hundreds of nm) inside the analyte medium, helps to make the sensor specific when a specific binder is used. SPR-based biosensors are able to detect biomolecular interactions directly with no need for labelling [53]. This characteristic allows real-time measurements of the reaction kinetics, analyte concentration, and binding parameters. The SPR is expressed by a dark line on a white background, which represents the SPR angle determined by the RI of the analyte. The shift in RI is measured by a camera and line detection algorithm integrated within the SPR device that identifies a change in pixels, which is then translated into a shift in RI based on the device pre-calibration (Figure 3). Detection of a large analyte (>1 kDa) is usually performed using a direct assay format [57]. In the direct detection method, the bio-specific capture entity (antibody) is attached to the sensor chip surface, thereby inducing a sensor response. The type of the metal film on the SPR chip where plasmon waves are generated is critical for sensitive SPR sensing [58]. In order to achieve high-sensitivity in SPR sensing, a silver substrate is a good option as plasmon coupling on silver substrates exhibits a sharper angular resonance, thus yielding a higher sensitivity [59]. However, silver has poor chemical stability and is susceptible to thermal desorption and oxidation [60], which precludes its wide use for SPR sensing. Therefore, gold is mostly used in the SPR chip as they possess stable chemical and optical properties [61]. Most SPR chips utilize a gold nano-layer for sensing [53,62,63]. In this study, a bi-metallic layer is used, consisting of a silver and gold chip to detect sub nano concentrations of the stroke markers (Figure 4) [49,64,65,66].

## 2. Materials and Methods

### 2.1. Equipment

PhotonicSys SPR H5 was purchased from PhotonicSys (Neveh Shalom-Wahat Alsalam, Israel; www.photonicsys.com) and was used for signal acquisition. A peristaltic pump Masterflex L/S was purchased from Cole-Parmer (Vernon Hills, IL, USA) and was used for washing the SPR gold chip while still integrated into the PhotonicSys SPR H5 system. An ultrasonic Cleaner SONICA model MH was purchased from Soltec (Milano, Italy) and was used for the cleaning of new SPR gold chips. Field Emission Scanning Electron Microscope (FE-SEM), model: Supra 55, was purchased from Carl Zeiss (Oberkochen, Germany) and was used to characterize the integrity of the SPR gold chip surface. The balance was purchased from Mettler Toledo (Columbus, OH, USA) and was used for the scaling of the chemicals.

### 2.2. Materials

3-aminopropyltriethoxysilane, 99% (APTES) (SKU 440140), glutaraldehyde, 25% in H2O (SKU G5882), phosphate buffer saline (PBS, pH 7.4) (SKU P4417), Tween 20 (SKU P7949), bovine serum albumin (BSA) (SKU 7030), 2-(N-Morpholino) ethanesulfonic acid (MES) sodium salt (SKU M5057), ethanolamine (EA) (≥ 99.0%) (SKU 398136), 11-Mercaptoundecanoic (MUA) acid, 95% (SKU 450561), absolute ethanol (SKU 02856), N-(3-Dimethylaminopropyl)-N′-ethylcarbodiimide hydrochloride (EDC) (SKU E1769), N-Hydroxysuccinimide,98% (NHS) (SKU 130672) were purchased from Sigma-Aldrich and were used for the functionalization of the SPR gold chip. Immunoreagents: NT-proBNP peptide (CAT#orb81959) and Anti-NT-proBNP 15F11 Antibody (CAT#orb79568) were purchased from Biorbyt and used for the development of the biosensor for NT-proBNP detection. S100β protein (CAT#8S9b) and Anti-S100β 8B10 Antibody (CAT#4S37) were purchased from HyTest and used for the development of the biosensor for S100β detection. SPR Substrate: SPR silver-gold substrates, which we refer to simply as “gold-chips”, were purchased from PhotonicSys.

### 2.3. Porcine Plasma

Detection validation was conducted with spiked porcine plasma as a medium to mimic human plasma, which is usually used for NT-proBNP and S100β diagnostic testing. With a Singapore Agri-Food & Veterinary Authority (AVA) official permit, porcine whole blood was purchased from Primary Industries Pte. Ltd. (Singapore). Firstly, to prevent blood coagulation, heparin solution was prepared by dissolving 100 mg heparin sodium salt in 20 mL 0.1 M Krebs-Ringer Phosphate buffer (pH 7.3) and then mixed with 1 L of the blood. The blood was then aliquoted to 50 mL falcon tubes and centrifuged for 30 min at 400× *g*. The plasma was later purified from red blood cell remains under the same separation condition. The aliquoted porcine plasma was stored frozen at −80 °C. Later, various concentrations of NT-proBNP and S100β were spiked into the porcine plasma for biosensor detection validation.

### 2.4. Device Calibration

The detection system was factory-calibrated and did not require any user intervention. However, there could be cases where calibration was lost. The device can then be recalibrated by measuring the pixel value of different concentrations of glycerol (5% *v*/*v*–25% *v*/*v*) diluted in deionized (DI)-water (Figure 3). Then, the pixel was analyzed according to the solution’s known RI value. DI water was used in the reference channel. The PhotonicSys SPR H5 recognizes the shift in RI through monitoring the shift in the dark line location in pixels. The shift in RI was measured by a camera and line detection algorithm integrated within the SPR device that identifies a change in pixels, which is then translated into a shift in RI based on the device pre-calibration.

### 2.5. Gold Chip Functionalization

The gold SPR chip was functionalized as presented in Figure 4. The gold chip was made from a bimetallic layer; the first layer was 30 nm silver and the top layer was 15 nm gold. The gold chip was first cleaned in an ultrasonic bath incubated with deionized (DI) water for 20 min at room temperature (RT) to remove inorganic and organic contaminants on the surface. Then, the chip was incubated in 1 mM MUA-ethanolic solution for 18 h at 4 °C in the dark. This was to enable the formation of a self-assembled monolayer with a carboxyl functional group on the surface [49,64,65,66]. Thereafter, the chip was soaked in an activation reagent solution (0.1 mM NHS - 0.4 mM EDC - 10 mM MES buffer pH 5) for 60 min at RT to activate the terminal carboxyl acid group [64,65,66]. After the activation of the carboxylic acid group, specific antibody (Anti-NT-proBNP or Anti-S100β) was immobilized on the chip surface by soaking the chip in 20 µg/mL antibody solution diluted in 0.05% (*v*/*v*) PBS-Tween20 for 18 h at 4 °C. Lastly, the chip was soaked in 50 mM Ethanolamine-PBS solution for 30 min at RT for blocking of non-specific binding sites. 

### 2.6. Biomarker Measurement

**Figure 5:**
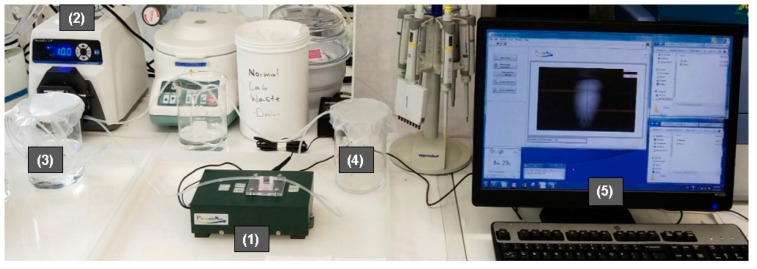
Surface plasmon resonance biosensor system setup. (1) PhotonicSys SPR H5; (2) peristaltic pump; (3) source solution; (4) drain solution; (5) detection software.

The surface plasmon resonance biosensor setup is shown in Figure 5. Firstly, the gold SPR chip was cleaned and then functionalized with the bio-specific capture entity (antibody), as described in Section 2.5. The prism was made of SF11 glass and the LD wavelength was 635 nm. Thereafter, the specific biomarker detection was done by passing different concentrations of analyte (biomarkers NT-proBNP or S100β) over the SPR gold chip. The biomarkers were either diluted in DI water or in spiked plasma samples. Lastly, the changes in RI values were recorded and analyzed. DI water was used in the reference channel.

### 2.7. Data Analysis

Data were collected in Microsoft Excel and then analyzed using SPSS 23.0 (IBM SPSS Statistics, version 23). Differences in the pixel and RI values were analyzed by descriptive analysis. Results are presented as the median value (the 50% percentile) with the corresponding interquartile range (IQR) of 25% to 75% percentiles. The limit-of-detection (LOD) for each measurement was determined based on the sensitivity and the average standard-deviation (SD), according to the following equation:

$$Limit-Of-Detection\left(LOD\right)=\frac{\left(k\times SD\right)}{m}$$

where *k* is the confidence level (*k* = 3), SD (refractive index units (RIU)) is the average SD for each specific measurement and m (RIU/(ng/mL)) is the calibration sensitivity (the slope of the linear plot for each specific measurement).

## 3. Results and Discussion

### 3.1. Gold Chip Functionalization

**Figure 6:**
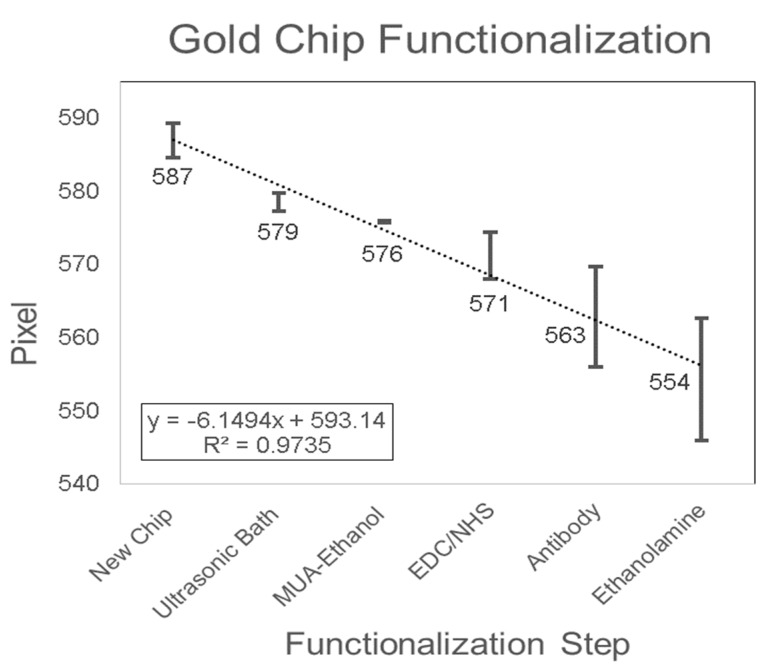
Gold SPR chip functionalization.

The SPR biosensor mechanism was based on the detection of biomarkers using a functionalized SPR chip. The SPR chip was functionalized with a bio-specific capture entity (antibody) acting as the biomolecular recognition element [49]. The functionalization of the gold SPR chip included the removal of inorganic and organic contaminants with the ultrasonic bath, cleaning and activation using MUA-ethanolic solution to allow the formation of a self-assembled monolayer with carboxyl functional group on the surface, coupling of the antibody onto the substrate surface with EDC/NHS solution, and blocking with ethanolamine. In order to validate the surface modification of the gold SPR chip, the pixel value of DI Water was monitored after each functionalization step (Figure 6). Adsorptions on the gold surface changed the SPR resonance angle θ_SPR_, and consequently the RI of the analyte near the gold layer [67], which resulted in a change in the pixel value measured in the SPR detection system. The pixel value decreased over each functionalization step with the linear equation Y = −6.1494X + 593.14 (R^2^ = 0.9735). The decrease in pixel value indicated that the SPR dip shifts up and that the SPR resonance angle θ_SPR_ increased. This positive shift in the SPR dip occurs when the adsorptive structure was applied on the chip [68]. Therefore, the change in surface chemistry on the chip was validated with the result shown in Figure 6. The results presented were in agreement with the findings from Asta Kausaite et al. [69], who found an increase in SPR resonance angle through the surface functionalization procedure with MUA, EDC/NHS, and Ethanolamine, and Omar et al. [70], who found an increase in SPR resonance angle with the introduction of EDC/NHS onto the chip layer during the immobilization procedure. In addition, these results were also in agreement with Karabchevsky et al. [67], who found an increase in SPR resonance angle following 11-MUA incubation and antibody immobilization on silver SPR chips. To conclude, the pixel negative shift, which is equivalent to an SPR dip positive shift, validated the change of surface chemistry on the gold SPR chip.

### 3.2. Biomarkers Measurement

**Figure 7:**
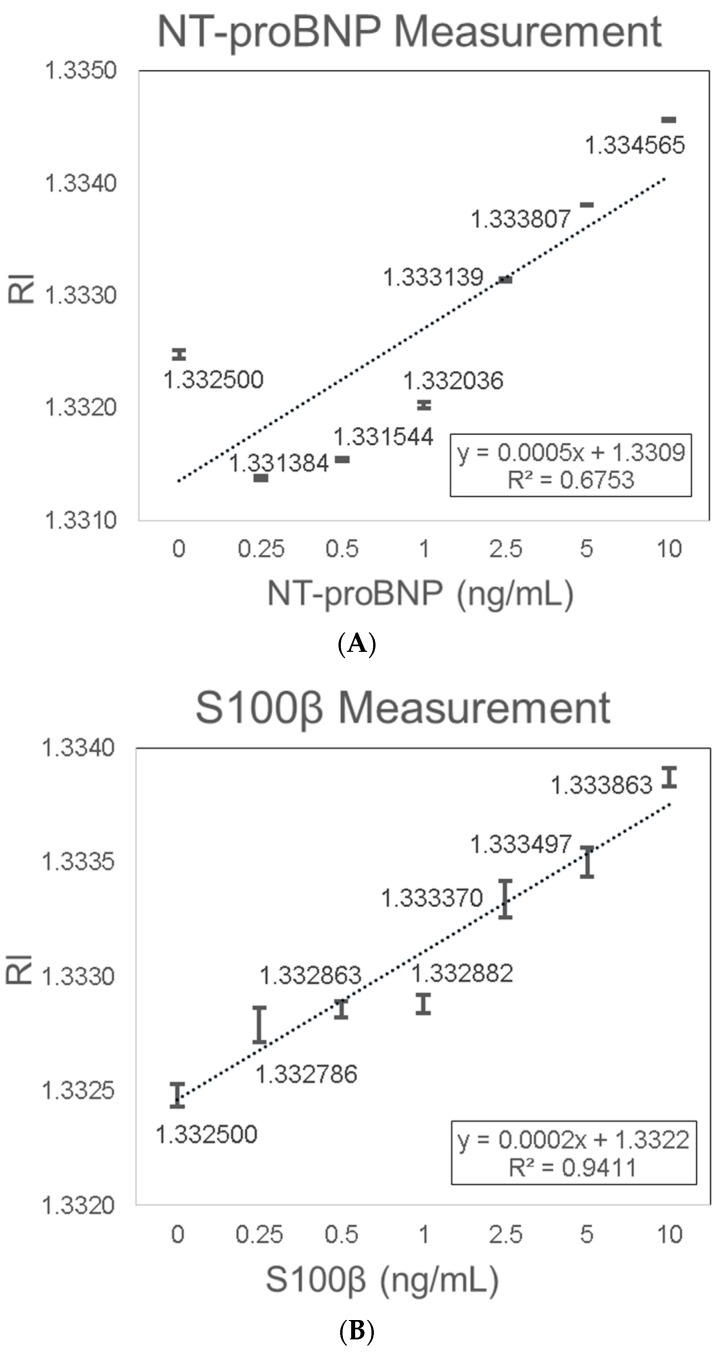
Stroke biomarkers measurement using bold SPR chip. (**A**) NT-proBNP; (**B**) S100β.

NT-proBNP and S100β were chosen as the two stroke biomarkers candidates as they have been extensively studied and have shown great diagnostic accuracy. Firstly, the gold SPR chips were functionalized with a specific antibody via an MUA-EDC/NHS-based reaction. After specific antibody functionalization, the chips were exposed to increasing biomarker concentrations between 0.25 ng/mL and 10 ng/mL diluted in DI water. NT-proBNP detection showed consistent results with a linear trend of increasing RI signal with increasing NT-proBNP concentrations. The increase in RI with increasing NT-proBNP concentrations was characterized with the linear equation Y = 0.0005X + 1.3309 (R^2^ = 0.6753) (Figure 7A). The LOD of 0.12 ng/mL NT-proBNP was determined based on the sensitivity (0.0005 (RIU/(ng/mL))) and the average SD (0.00002 (RIU)). It should be noted that the first data point corresponding to zero concentration deviates from the rest of the data points, possibly because the protein can interact slightly with the uncoated sites of the gold surface. Even if we ignore this point, the LOD will improve further because the slope will increase (higher sensitivity). NT-proBNP sensing was previously explored by Binbin Luo et al. [71], with a label-free immunosensor platform based on excessively tilted fiber gratings (Ex-TFGs). The lowest detectable concentration of 0.5 ng/mL for NT-proBNP was obtained. Yuji Teramura et al. [72] explored the detection of BNP (NT-proBNP is the derivative of BNP) using an SPR biosensor. The SPR signal was amplified by using a sandwich biosensor layout with a secondary antibody conjugated with streptavidin nanobeads. By this method, the SPR signals were intensified and sub-nano (0.25 ng/mL) concentrations of BNP could thus be detected. Hye Ri Jang et al. [73] also investigated the detection of BNP using SPR sandwich biosensor layout, but with the use of aptamer as the bio-specific capture entity. Kurita et al. [74] also explored BNP detection using a microfluidic device combined with a portable SPR sensor system. The sensor achieved a detectable concentration range of 5 pg/mL–100 ng/mL by monitoring the SPR angle shift.

The functionalized gold SPR chips were also functionalized with a specific anti-S100β antibody and then exposed to increasing S100β concentrations. S100β detection showed consistent results with a linear trend of increased RI signal with increasing S100β concentrations. The increase in RI with increasing S100β concentrations was characterized by the linear equation Y = 0.0002X + 1.3322 (R^2^ = 0.9411) (Figure 7B). The LOD of 0.75 ng/mL S100β in water was determined based on the sensitivity (0.0002 (RIU/(ng/mL))) and the average SD (0.00005 (RIU)). Previously, SPR technology was utilized for the testing of protein–protein interactions of S100β. Harald Seitz et al. [75] showed that by using SPR technology and functional protein microarrays, specific calcium-dependent interactions between Arg-Gly-Ser-(RGS)-His_6_ tagged proteins and GST-tagged S100B could be detected. In a different study by Estelle Leclerc [76], SPR technology was utilized to examine the interaction between S100 proteins and isolated domains of the receptor for advanced glycation end products (RAGE), in particular, the measurement of the binding of S100B to the RAGE V domain. Our results demonstrated for the first time a sensitive and label free SPR biosensor for the detection of NT-proBNP and S100β. 

### 3.3. Validation in Spiked Porcine Plasma Samples

**Figure 8:**
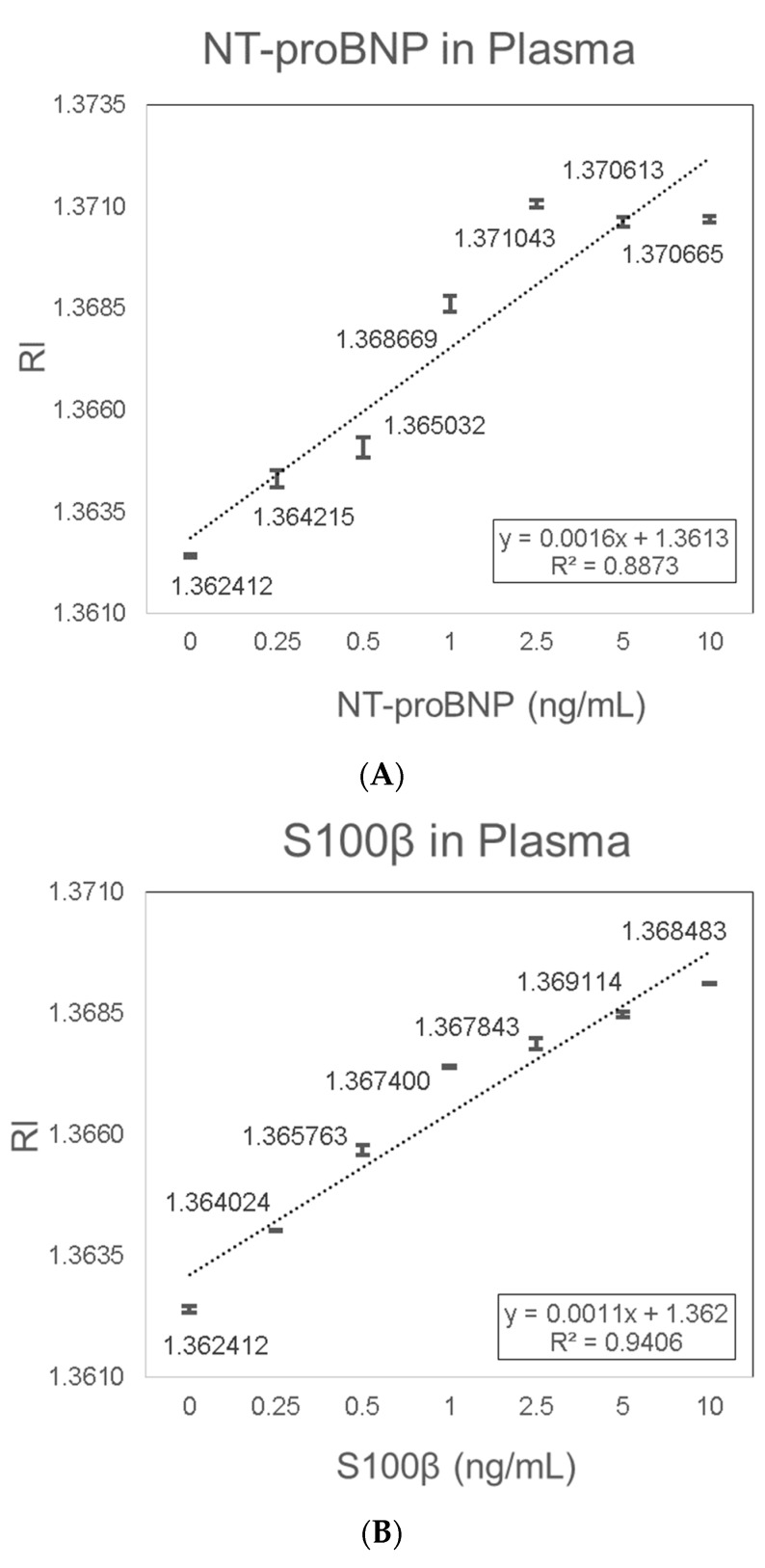
Validation in spiked plasma samples. (**A**) NT-proBNP; (**B**) S100β.

Porcine plasma was used as a medium to mimic the sample environment of human plasma, which is the preferred sample type for diagnostic testing of NT-proBNP and S100β. NT-proBNP detection in plasma showed consistent results with a linear trend of increased RI signal with increasing NT-proBNP concentrations. The increase in RI with increasing NT-proBNP concentrations was characterized by the linear equation Y = 0.0016X + 1.3613 (R^2^ = 0.8873) (Figure 8A). The LOD of 0.075 ng/mL NT-proBNP in plasma was determined based on the sensitivity (0.0016 (RIU/(ng/mL))) and the average SD (0.00004 (RIU)). S100β detection in plasma also showed consistent results with a linear trend of increased RI signal with increasing S100β concentrations. The increase in RI with increasing S100β concentrations was characterized by the linear equation Y = 0.0011X + 1.362 (R^2^ = 0.9406) (Figure 8B). The LOD of 0.136 ng/mL S100β in plasma was determined based on the sensitivity (0.0011 (RIU/(ng/mL))) and the average SD (0.00005 (RIU)).

## 4. Conclusions

In this study, a functionalized gold SPR chip was developed for the specific detection of stroke biomarkers using an SPR POC device. This was the first time SPR technology had been utilized as a POC device for the detection of blood-based biomarkers. NT-proBNP and S100β were chosen as the two stroke biomarkers candidates as they have been extensively studied and have shown great diagnostic accuracy. The SPR biosensor mechanism was based on the detection of biomarkers using a functionalized SPR chip with a bio-specific capture entity (antibody) acting as the biomolecular recognition element [49]. The biomolecular interaction of the specific binding of the analyte is detected as a RI shift. Gold substrates are mostly used for SPR chips as they possess stable chemical and optical properties [53,61,62,63]. Firstly, the gold SPR chips were functionalized with a specific antibody via a MUA-EDC/NHS-based reaction. After specific antibody functionalization, the chips were exposed to increasing biomarker concentrations between 0.25 ng/mL and 10 ng/mL. The shift in RI was measured by a camera. NT-proBNP detection in water showed consistent results with a linear trend of increased RI signal with increasing NT-proBNP concentrations. The increase in RI with increasing NT-proBNP concentrations was characterized by the linear equation Y = 0.0005X + 1.3309 (R^2^ = 0.6753). The LOD of 0.12 ng/mL NT-proBNP was determined based on the sensitivity (0.0005 (RIU/(ng/mL))) and the average SD (0.00002 (RIU)). In addition, NT-proBNP detection in plasma showed consistent results with a linear trend of increased RI signal with increasing NT-proBNP concentrations; Y = 0.0016X + 1.3613 (R2 = 0.8873). The LOD of 0.075 ng/mL NT-proBNP in plasma was determined based on the sensitivity (0.0016 (RIU/(ng/mL))) and the average SD (0.00004 (RIU)). Moreover, S100β detection in water showed consistent results with a linear trend of increased RI signal with increasing S100β concentrations. The increase in RI with increasing S100β concentrations was characterized by the linear equation Y = 0.0002X + 1.3322 (R^2^ = 0.9411). The LOD of 0.75 ng/mL S100β in water was determined based on the sensitivity (0.0002 (RIU/(ng/mL))) and the average SD (0.00005 (RIU)). In addition, S100β detection in plasma showed consistent results with a linear trend of increased RI signal with increasing S100β concentrations; Y = 0.0011X + 1.362 (R^2^ = 0.9406). The LOD of 0.136 ng/mL S100β in plasma was determined based on the sensitivity (0.0011 (RIU/(ng/mL))) and the average SD (0.00005 (RIU)). The RI of blank plasma samples was 1.362412, and the lowest concentration tested for both biomarkers showed a high shift in the RI signal for (0.25 ng/mL NT-proBNP (1.364215) and S100β (1.364024)). To conclude, the detection was tested with water and plasma samples. NT-proBNP and S100β were detected in a range of concentrations for stroke, from 0.25 ng/mL to 10 ng/mL. The biosensor demonstrated a clinically relevant limit-of-detection of less than ng/mL.
